# Nonbacterial Thrombotic Endocarditis and Mechanical Valve Thrombosis in a Patient with Antiphospholipid Syndrome

**DOI:** 10.1016/j.case.2025.12.002

**Published:** 2026-02-09

**Authors:** Fox Bravo, Christy Lougheed, Jeffery S. Tran, Kwan S. Lee, Toshinobu Kazui, Arka Chatterjee, Tushar Acharya

**Affiliations:** a College of Medicine, University of Arizona, Tucson, Arizona; b Sarver Heart Center, University of Arizona, Tucson, Arizona; c Division of Cardiology, Washington University Barnes-Jewish Hospital, St. Louis, Missouri; d Division of Cardiology, Mayo Clinic, Scottsdale, Arizona

**Keywords:** Antiphospholipid syndrome, Nonbacterial thrombotic endocarditis, Mchanical valve thrombosis, Transesophageal echocardiography, Fibrinolytic therapy

## Abstract

• NBTE causing severe MS is extremely rare (<2% of APS patients).
• MVT developed 2 months postop with subtherapeutic INR.
• Low-dose tPA successfully treated valve thrombosis without bleeding complications.
• Enhanced anticoagulation strategy (INR 3-4) prevented recurrent thrombosis.
• Fibrinolytic therapy avoided high-risk redo cardiac surgery in APS patient.

## Graphical abstract

**Figure:**
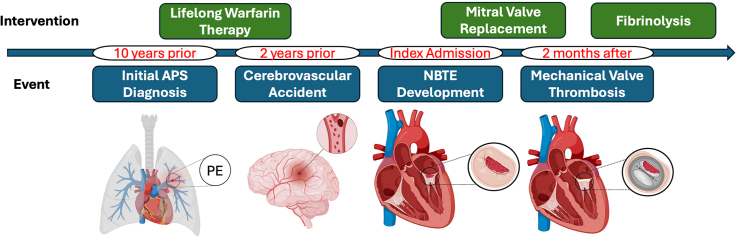

## Introduction

We present a case of antiphospholipid syndrome– (APS-) associated nonbacterial thrombotic endocarditis (NBTE) causing severe mitral stenosis (MS) requiring surgical valve replacement, followed by mechanical valve thrombosis (MVT) successfully treated with fibrinolytic therapy. This case brings forth multiple APS-associated thrombotic complications including pulmonary embolism (PE), stroke, NBTE-induced MS (which is exceedingly rare), and MVT, thus stressing the difficulties in managing thrombosis risk despite oral anticoagulation therapy. It demonstrates the utility of multimodality imaging in managing these complications and highlights the successful use of echocardiography-guided fibrinolytic therapy as a viable alternative to high-risk redo surgery, providing a potential treatment pathway for similar patients with recurrent thrombotic complications.

## Case Presentation

A 45-year-old patient with primary APS on warfarin presented to an outside hospital with a 2-month history of hemoptysis. Pertinent past medical history included an unprovoked PE 10 years earlier and a cerebrovascular accident 2 years earlier despite oral anticoagulation therapy with warfarin. Chest computed tomography (CT) was interpreted as multifocal pneumonia, and transthoracic echocardiography (TTE) showed a possible vegetation on the mitral valve (MV). The patient was started on systemic antibiotics for presumptive diagnoses of infective endocarditis (IE) and pneumonia. Due to progressive hypoxemia and hemoptysis despite antibiotics and concern for severe MS, the patient was transferred to our center for tertiary care.

On arrival, heart rate was 80 beats per minute (bpm), blood pressure was 121/75 mm Hg, and pulse oximetry showed a saturation of 92% on 5 L/min of supplemental oxygen. On examination the patient looked comfortable.

**Figure 1:**
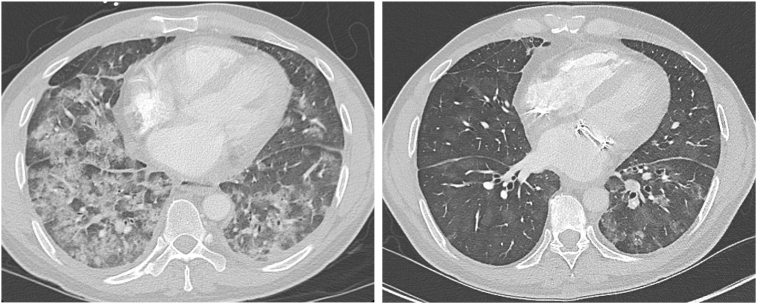
Chest CT, axial display with contrast (lung windows), concerning for DAH (*left*) from MS and its subsequent resolution after MV replacement (*right*).

Chest CT with contrast at our institution ruled out PE and was thought to be more suggestive of diffuse alveolar hemorrhage (DAH) from elevated pulmonary venous pressures from MS (Figure 1). The diagnosis of DAH was confirmed through bronchoscopy-assisted bronchoalveolar lavage showing copious blood-tinged secretions. Lavage cultures were negative for infectious pathogens.

**Figure 2:**
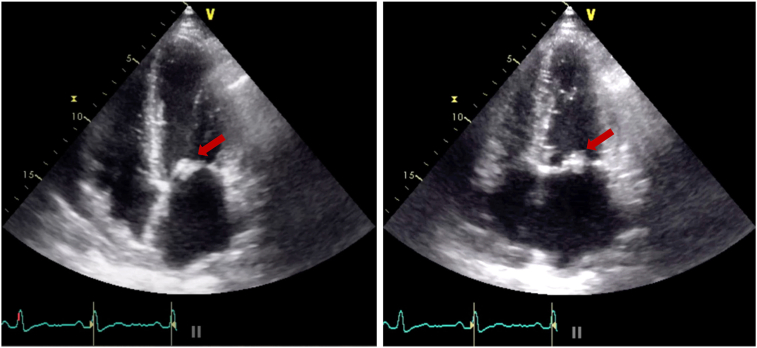
Two-dimensional TTE, apical 4-chamber diastolic (*left*) and systolic (*right*) views, demonstrates normal left ventricular size and function and a large echogenic mass (*arrows*) attached to the PMVL causing restricted leaflet mobility.

**Figure 3:**
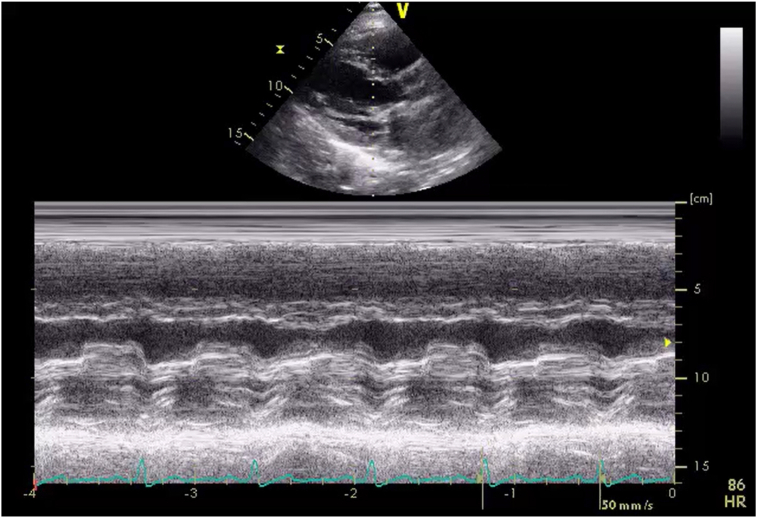
M-mode TTE, parasternal long-axis view, demonstrates a markedly reduced diastolic excursion of the mitral leaflets, reduced E-F slope consistent with MS, and a mass obscuring the mitral orifice.

Two-dimensional (2D) TTE at our institution demonstrated a large echodensity on the posterior MV leaflet (PMVL) with restricted leaflet mobility (Figure 2, Video 1), mitral obstruction on M-mode and 2D imaging (Figure 3, Video 2), and a mean diastolic mitral gradient of 21 mm Hg at a heart rate of 91 bpm. The MV area by pressure half time was 1.3 cm^2^, and by continuity equation it was 0.8 cm^2^. Left ventricular ejection fraction was calculated at 67% by biplane Simpson method.

**Figure 4:**
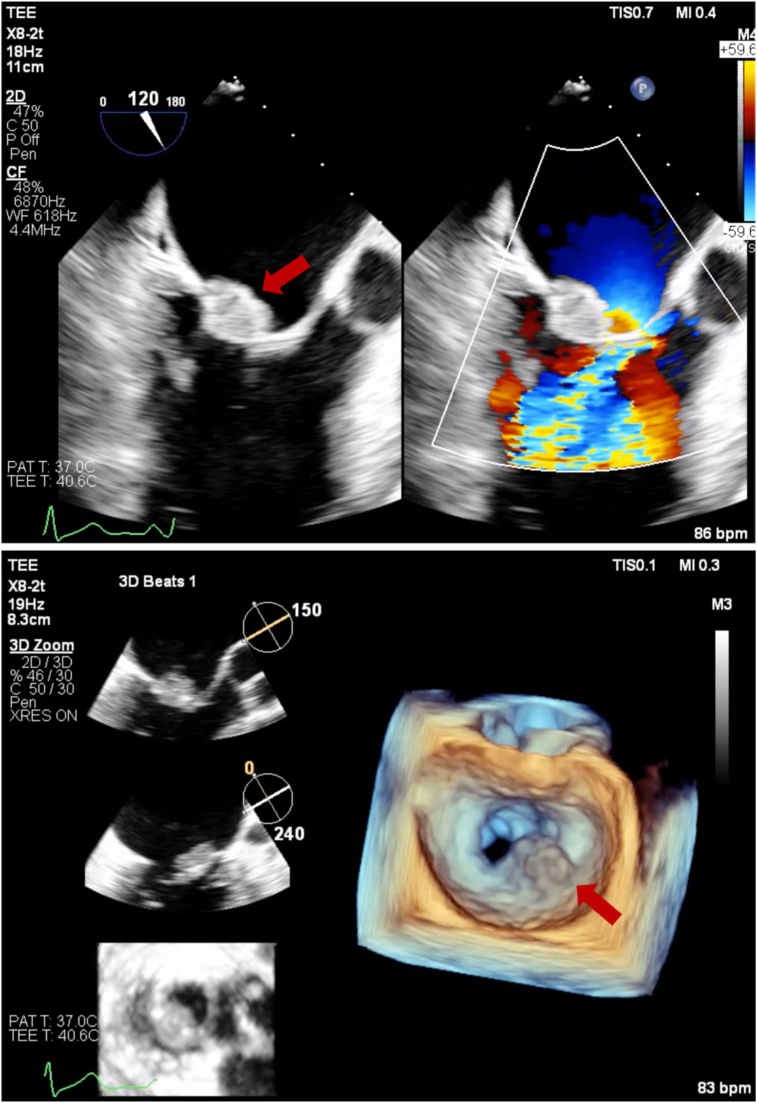
Two-dimensional TEE, mid-esophageal zoomed long-axis (120°) diastolic views (*top panel*) without (*left*) and with (*right*) color-flow Doppler, and 3D TEE (*bottom panel*) with multiplanar diastolic reconstruction displays in orthogonal long-axis orientation (*top, middle*) and short-axis orientation (*bottom*) and volume-rendered diastolic reconstruction (*right*), demonstrate a 1.9 × 1.8 cm vegetation localized to the P2 scallop of the PMVL (*arrows*) causing restricted opening and flow obstruction.

**Figure 5:**
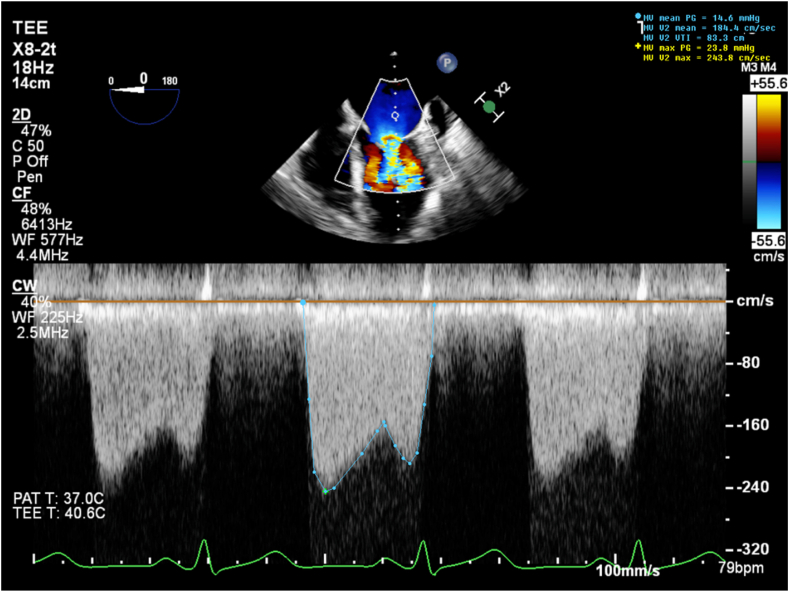
Two-dimensional TEE, mid-esophageal long-axis (0°) diastolic view with continuous-wave Doppler cursor positioned across the MV, demonstrates an abnormal spectral Doppler profile with severely elevated transmitral velocities consistent with a mean diastolic gradient of 15 mm Hg at 79 bpm and severe MS.

Three-dimensional (3D) transesophageal echocardiography (TEE) confirmed a 1.9 × 1.8 cm vegetation on the P2 scallop of the PMVL causing restricted MV opening (Figure 4, Videos 3 and 4). The mass was broad-based, sessile, echobright, well-circumscribed with irregular margins, and attached to the atrial aspect of the PMVL. It extended from the mid-leaflet to the tip without causing any leaflet destruction. Continuous-wave Doppler confirmed severe MS with a mean gradient of 15 mm Hg at 79 bpm (Figure 5). There was partial fusion of the medial scallops (A2/P2-A3/P3) and thickening of the subvalvular apparatus causing color flow aliasing and contributing to stenotic gradients. Morphology and location of the mass, without leaflet destruction, along with the history of APS, absence of septic clinical picture, and negative cultures, were all suggestive of NBTE.

**Figure 6:**
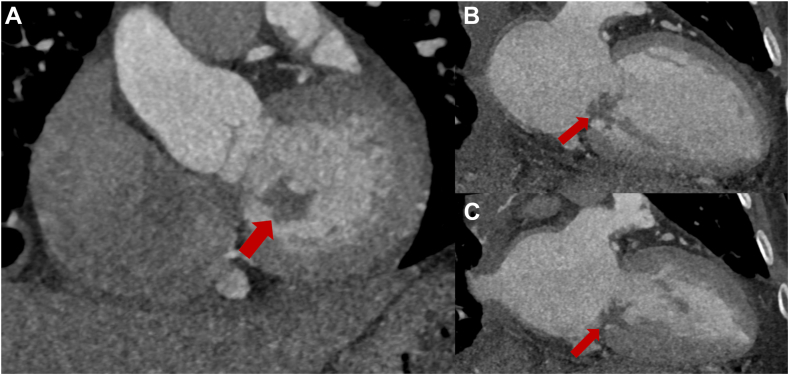
Cardiac CT, multiplanar reconstruction, oblique sagittal (**A**, basal cardiac short axis), oblique coronal long axis (cardiac 2-chamber view) in diastole **(B)** and systole **(C)**, demonstrates normal left ventricular size and function and the irregular-shaped, large mass (vegetation) attached to the PMVL.

Cardiac CT (CCT) demonstrated normal coronaries and an irregular mass on the PMVL consistent with a vegetation (Figure 6).

Laboratory studies revealed persistently positive antiphospholipid antibodies: lupus anticoagulant (LA) positive by dilute Russell's viper venom time, anticardiolipin IgG 112 GPL units (normal <20), and anti-β2-glycoprotein I IgG 58 SGU (normal <20). Complement levels were normal (C3, 97 mg/dL; C4, 7 mg/dL).

Right heart catheterization demonstrated elevated pulmonary artery and mean capillary wedge pressures of 59/18 mm Hg and 26 mm Hg, respectively, with a pulmonary vascular resistance of 2.17 Wood units. Cardiac output was normal at 6.4 L/min, and cardiac index at 3.37 L/min/m^2^. These findings suggested pulmonary venous hypertension.

**Figure 7:**
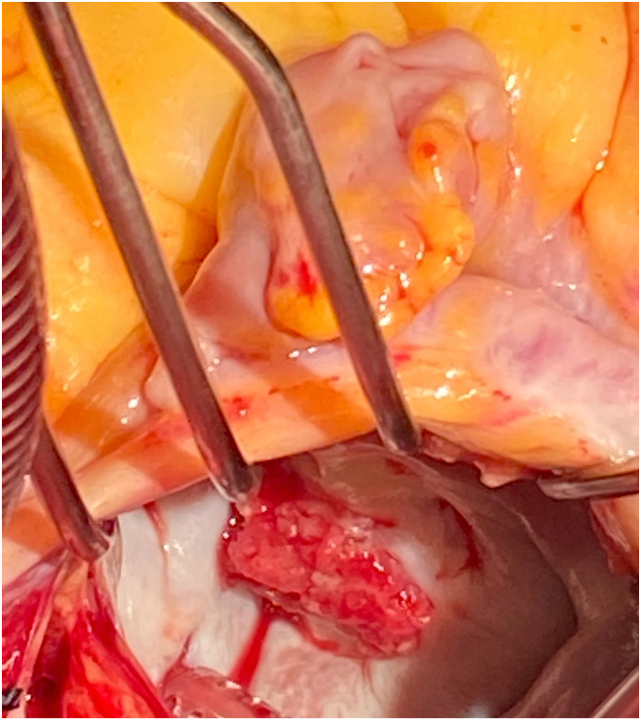
Intraoperative photograph demonstrates the large vegetation attached to the PMVL that correlates well with the findings on noninvasive diagnostic imaging.

The case was discussed in the multidisciplinary heart team meeting. Given the young age and need for systemic anticoagulation for APS, mechanical MV replacement was deemed to be the most appropriate option. The patient underwent MV and subvalvular apparatus resection (Figure 7) with placement of a 25/33 mm On-X mechanical valve. Pathology of the valvular vegetation demonstrated fibrotic and thrombotic components consistent with NBTE.

Apart from paroxysmal postoperative atrial fibrillation requiring cardioversion, the remainder of the immediate postoperative course was uncomplicated. The patient was discharged on warfarin with an international normalized ratio (INR) goal of 2.5 to 3.5.

**Figure 8:**
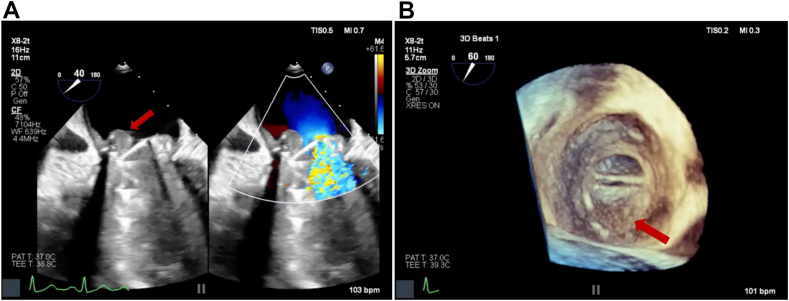
Two-dimensional TEE, mid-esophageal long-axis (40°) diastolic views **(A)** without (*left*) and with (*right*) color-flow Doppler, and **(B)** 3D TEE, short-axis (60°) volume-rendered reconstruction demonstrate a large mass (*arrow*, thrombus) on the mechanical MV restricting the opening of the posterior-facing mechanical disk (a shadowing artifact is also seen within the left ventricle beyond the prosthetic valve).

**Figure 9:**
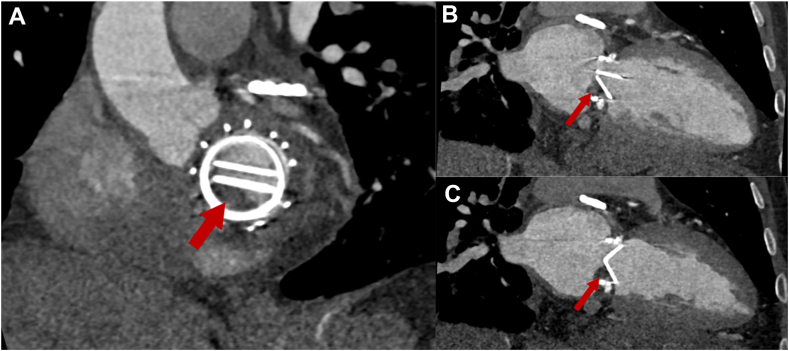
Cardiac CT, multiplanar reconstruction, oblique sagittal (**A,** basal cardiac short axis), and oblique coronal long axis (cardiac 2-chamber view) displays in diastole **(B)** and systole **(C)**, demonstrates normal left ventricular size and function, the mechanical MV, and a large thrombus (*arrow*) restricting the opening of the posterior-facing mechanical disk.

The patient did well in the next 2 months with resolution of chest CT changes of DAH (Figure 1) but then noted a return of progressive dyspnea and hemoptysis after a period of subtherapeutic INR (1.7-2.1). A TTE showed elevated mechanical MV mean gradients, while chest CT showed recurrence of DAH. Follow-up TEE demonstrated a large 1.2 × 1.1 cm, irregular, sessile echodensity on the mechanical MV precluding the opening of the posterior-facing tilting disk, although closure was unrestricted (Figure 8, Videos 5 and 6). This was most consistent with MVT. Thrombus restricted mechanical disk opening was confirmed on CCT (Figure 9). Mean mitral gradient was elevated at 15 mm Hg at a heart rate of 104 bpm, essentially replicating the previous MS pathophysiological presentation.

**Figure 10:**
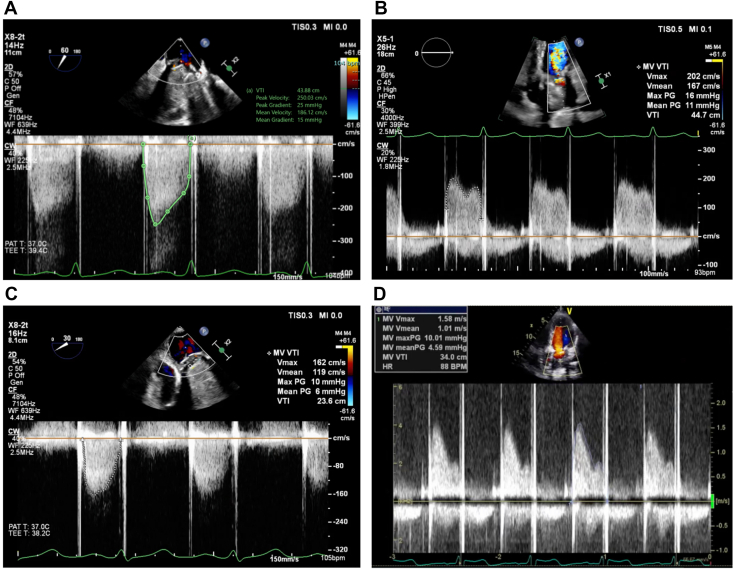
Serial 2D TEE- **(A, C)** and 2D TTE- **(B, D)** guided continuous-wave spectral Doppler profile displays of mean MV gradient over the course of fibrinolytic therapy representing the baseline findings (**A**, 15 mm Hg, 104 bpm), day 4 of treatment (**B**, 11 mm Hg, 93 bpm), day 10 of treatment (**C**, 6 mm Hg, 105 bpm), and day 14, end of fibrinolytic treatment (**D**, 5 mm Hg, 88 bpm).

The patient was admitted to the hospital and started on intravenous heparin. A multidisciplinary heart team meeting was held, and redo MV replacement versus medical therapy were discussed in detail with the patient for shared decision-making. It was felt that a redo surgery would not preclude a risk of recurrence given the persistently elevated thrombotic risk with APS. A long-term viable medical strategy was needed. After an extensive discussion of the risks and benefits involved with either intervention, the patient elected to pursue fibrinolytic therapy. A low-dose tissue plasminogen activator (tPA) protocol was initiated. Five cycles of tPA were administered at 1.0 mg/h for 25 hours. After each cycle, the patient was placed on therapeutic heparin for 6 hours, and echocardiography was performed to evaluate valve function. Each cycle of tPA caused a progressive decrease in the MV gradient, with final imaging via TEE demonstrating a mean mitral gradient of 6 mm Hg at 105 bpm (Figure 10). The patient experienced improvement in symptoms without any bleeding complications and was subsequently discharged with warfarin to a goal INR of 3 to 4 (per multidisciplinary consultation). The patient was also prescribed therapeutic enoxaparin to be self-administered for INR less than 3.0.

Repeat TTE performed 2 weeks post–fibrinolytic therapy demonstrated an MV diastolic gradient of 5 mm Hg at a heart rate of 87 bpm. The patient reported no subsequent symptoms of hemoptysis or shortness of breath.

## Discussion

Nonbacterial thrombotic endocarditis is rare, with an unknown incidence, and is associated with procoagulant states like malignancy, systemic lupus erythematosus, and APS.^1^ Heart valve lesions such as vegetations, valve thickening, and valvular dysfunction are frequent with NBTE and are seen in about one-third of patients with APS. The MV is most commonly affected, followed by the aortic valve (62% and 24%, respectively) according to a recent case series.^1^, ^2^, ^3^ Mitral regurgitation is the predominant valvular abnormality, affecting 26% to 46% of patients, whereas stenosis, as seen in this report, is rare and has been documented in <2%.^1^^,^^2^^,^^4^ In patients with valve replacement, MVT can be a serious complication (incidence, 0.5%-8%).^3^^,^^5^^,^^6^

This case demonstrates several important teaching points regarding the management of APS-associated valvular complications. First, NBTE-induced stenosis occurs through leaflet scarring, thickening, and thrombosis, like that seen in rheumatic valve disease. While anticoagulation remains the cornerstone of thrombosis and embolic prevention in APS, emerging evidence suggests it provides incomplete protection, with 54% to 70% of anticoagulated patients still experiencing embolic events and up to 36% developing new valvular lesions over time while being on therapeutic anticoagulation. This highlights the dual thrombotic and immune-inflammatory pathogenesis that anticoagulation alone cannot fully address.^7^^,^^8^ Systemic anticoagulation is used in the secondary prevention of NBTE-related embolic strokes, while surgical valve replacement is typically the treatment of choice for severe valvular stenosis or regurgitation.

Multimodal cardiovascular imaging played a central role in both the diagnosis and management of this complex case. The distinction between NBTE and IE can be challenging as both present with valve vegetations and valvular dysfunction. However, several features on echocardiography favored NBTE in our patient. The vegetation was immobile, sessile, and broad-based. It did not cause any valve destruction favoring the diagnosis of NBTE.^7^ Three-dimensional TEE was particularly valuable in our case, allowing precise localization of the vegetation to the P2 scallop and visualization of the fused leaflet tips. Three-dimensional imaging provided spatial information critical for surgical planning and confirming the extent of valvular and subvalvular involvement.

The integration of echocardiography with other imaging modalities enhanced diagnostic accuracy throughout this patient's hospital course. Chest CT (along with bronchoalveolar lavage) assisted with identification of DAH secondary to elevated pulmonary venous pressures, providing the mechanistic link between MS and respiratory symptoms. Right heart catheterization confirmed pulmonary hypertension, corroborating echocardiographic severity assessment. Cardiac CT offered additional value in confirming vegetation initially and MVT postoperatively. This multimodality approach integrating TTE for screening, TEE for more detailed morphologic assessment with 3D rendering, and CT for confirmation was crucial for guiding treatment in this patient. Further, serial echocardiography after each tPA cycle was paramount to following mitral gradients and documenting MVT resolution.

Other imaging modalities worth considering are cardiovascular magnetic resonance for tissue characterization of valve masses, although its value in small, mobile lesions may be limited due to lower spatial resolution and volume averaging. Fluorodeoxyglucose positron emission tomography could potentially distinguish the IE from NBTE lesions as infective lesions tend to be more avid on positron emission tomography.

Due to its rarity, there are no guidelines regarding how to safely anticoagulate patients after surgical valve replacement in the setting of APS. Based on our experience, close perioperative anticoagulation monitoring is paramount, as patients are at high risk of thrombotic and hemorrhagic complications.^9^^,^^10^ An aggressive long-term postoperative anticoagulation strategy is imperative to prevent MVT and failure.^6^ The INR goal in the setting of mechanical valve prosthesis with APS is unclear, and existing guidelines are not specific to this subgroup of patients.^9^ International normalized ratio–guided anticoagulation is further complicated with LA autoantibody, which artificially elevates the INR in many commonly used assays.^11^ The chromogenic factor X assay can be used to calculate a “true” INR in the presence of LA autoantibodies but may not be readily available.^12^ Another alternative is to raise the INR goal, as was done in this case.

Management of acute MVT requires urgent intervention with either surgical valve replacement or thrombolytics. Per the 2020 American College of Cardiology/American Heart Association valve guidelines, the decision for medical versus surgical therapy can be determined by size of the thrombus (>0.8 cm^2^ favors surgery) and symptom severity, both of which would have pushed our patient toward surgical management. However, it was felt that redo surgery with similar medical management would likely result in recurrent MVT; therefore, we opted for thrombolysis followed by a more aggressive anticoagulation strategy. Repetitive low-dose, continuous infusion of fibrinolytics has demonstrated >90% hemodynamic success with <2% embolic or major bleeding events.^12^ The use of thrombolytics in our patient was efficacious in reducing clot burden, improving mean mitral gradient, and resolving pulmonary congestion–related symptoms.

Our case highlights the importance of maintaining therapeutic anticoagulation in APS patients with mechanical valves and demonstrates that fibrinolytic therapy can be a viable alternative to high-risk redo surgery when combined with enhanced anticoagulation strategies.

## Conclusion

Severe MS is a rare but potentially life-threatening presentation of APS-associated NBTE. Multimodality imaging with TTE, TEE, and CCT can help with the diagnosis, severity assessment, and surgical planning. Mechanical valves are preferred over bioprosthetic valves for this indication. Close monitoring of INR is essential to avoid subsequent MVT from APS-related hypercoagulability. If a thrombus should form on the mechanical valve, it can be successfully treated with echocardiography-guided low-dose fibrinolytic therapy as an alternative to redo MV replacement.

## Ethics Statement

The authors declare that the work described has been carried out in accordance with The Code of Ethics of the World Medical Association (Declaration of Helsinki) for experiments involving humans.

## Consent Statement

The authors declare that since this was a non-interventional, retrospective, observational study utilizing de-identified data, informed consent was not required from the patient under an IRB exemption status.

## Funding

The authors declare that this report did not receive any specific grant from funding agencies in the public, commercial, or not-for-profit sectors.

## Disclosure Statement

The authors reported no actual or potential conflicts of interest relative to this document.

## Supplementary Data

Video 1 Two-dimensional TTE, apical 4-chamber view, demonstrates normal left ventricular size and function and a large echogenic mass attached to the PMVL causing restricted leaflet mobility.

Video 2 Two-dimensional TTE, parasternal long-axis view, demonstrates normal left ventricular size and function, markedly reduced diastolic excursion of the mitral leaflets, and a mass obscuring the mitral orifice consistent with functional MS.

Video 3 Two-dimensional TEE, mid-esophageal zoomed long-axis (90°) view without (*left*) and with (*right*) color-flow Doppler, demonstrates a 1.9 × 1.8 cm vegetation localized to the P2 scallop of the PMVL causing restricted opening and flow obstruction.

Video 4 Three-dimensional TEE, with multiplanar reconstruction displays (*left*) in orthogonal long-axis (*top, middle*) and short-axis orientation (*bottom*) and volume-rendered short axis reconstruction (*right*) surgeon's view, demonstrates a large vegetation localized to the P2 scallop of the PMVL causing restricted opening and functional MS.

Video 5 Two-dimensional TEE, mid-esophageal long-axis (40°) view, without (*left*) and with (*right*) color-flow Doppler, demonstrates a large mass (thrombus) on the mechanical MV restricting the opening of the posterior-facing mechanical disk (a shadowing artifact is also seen within the left ventricle beyond the prosthetic valve).

Video 6 Three-dimensional TEE, short-axis volume-rendered reconstruction from the perspective of the left atrium, demonstrates a large mass (thrombus) on the mechanical MV restricting the opening of the posterior-facing mechanical disk.

## References

[1] Zmaili M.A., Alzubi J.M., Kocyigit D., Bansal A., Samra G.S., Grimm R. (2021). A contemporary 20-year cleveland clinic experience of nonbacterial thrombotic endocarditis: etiology, echocardiographic imaging, management, and outcomes. Am J Med.

[2] Hojnik M., George J., Ziporen L., Shoenfeld Y. (1996). Heart valve involvement (Libman-Sacks endocarditis) in the antiphospholipid syndrome. Circulation.

[3] Kolitz T., Shiber S., Sharabi I., Winder A., Zandman-Goddard G. (2019). Cardiac manifestations of antiphospholipid syndrome with focus on its primary form. Front Immunol.

[4] Perez-Villa F., Font J., Azqueta M., Espinosa G., Pare C., Cervera R. (2005). Severe valvular regurgitation and antiphospholipid antibodies in systemic lupus erythematosus: a prospective, long-term, followup study. Arthritis Rheum.

[5] Chamsi-Pasha M.A., Alyousef T., Sayyed S. (2014). Bioprosthetic mitral valve thrombosis complicating antiphospholipid antibody syndrome, successfully treated with thrombolysis. Echocardiography.

[6] Gürsoy M.O., Kalçık M., Yesin M., Karakoyun S., Bayam E., Gündüz S. (2016). A global perspective on mechanical prosthetic heart valve thrombosis: diagnostic and therapeutic challenges. Anatol J Cardiol.

[7] Ahmed O., King N.E., Qureshi M.A., Choudhry A.A., Osama M., Zehner C. (2024). Non-bacterial thrombotic endocarditis: a clinical and pathophysiological reappraisal. Eur Heart J.

[8] Venepally N.R., Arsanjani R., Agasthi P., Wang P., Khetarpal B.K., Barry T. (2022). A new Insight into nonbacterial thrombotic endocarditis: a systematic review of cases. Anatol J Cardiol.

[9] Gorki H., Malinovski V., Stanbridge R.D. (2008). The antiphospholipid syndrome and heart valve surgery. Eur J Cardiothorac Surg.

[10] Zuily S., Huttin O., Mohamed S., Marie P.Y., Selton-Suty C., Wahl D. (2013). Valvular heart disease in antiphospholipid syndrome. Curr Rheumatol Rep.

[11] Rosborough T.K., Shepherd M.F. (2004). Unreliability of international normalized ratio for monitoring warfarin therapy in patients with lupus anticoagulant. Pharmacotherapy.

[12] Özkan M., Gündüz S., Gürsoy O.M., Karakoyun S., Astarcıoğlu M.A., Kalçık M. (2015). Ultraslow thrombolytic therapy: a novel strategy in the management of PROsthetic MEchanical valve Thrombosis and the prEdictors of outcomE: the Ultra-slow PROMETEE trial. Am Heart J.

